
JOURNAL INFORMATION
==============================
NLM Title Abbreviation: Halls J Health
NLM Journal ID: 0011644

Hall's Journal of Health

Publisher: Biomedical Journal Digitization Project

ARTICLE INFORMATION
==============================
PMCID: PMC9191452
PMID: 36487166
Article version: 1
Subjects: Articles

The Old School-House

Howell Grandmother
Print publication date: 1868 Jul
Volume: 15
Issue: 7
First page: 157
Last page: 158
License: This work is free of known copyright restrictions. For more information, please see PMC Back Issue Digitization.
License URL: https://creativecommons.org/publicdomain/mark/1.0/

==============================

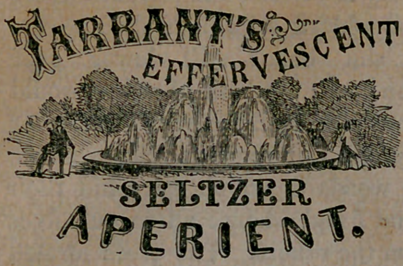

"AMER. SCHOOL INST.," FOUNDED 1855, Is a reliable Educational Bureau,

To aid all who seek well-qualified Teachers;
To represent Teachers who seek positions;
To give parents information of good schools;
To sell, rent, and exchange school properties.

Thirteen years' trial has proved the AMERICAN SCHOOL INST. a useful and efficient auxiliary in securing

"The Right Teacher for the Right Place."

Those who want Teachers should have the "Teachers' Bulletin," published in the AMERICAN EDUCATIONAL MONTHLY. Those who want positions should have the "Application Form"

Circulars explaining plan, and giving *Testimony* from first-class educational and business men, sent when asked for.

**J. W. SCHERMERHORN, A. M.,** Actuary,
14 Bond St., New York.

*Branch Offices in San Francisco Chicago and Boston.*

**Forestall Summer Fevers** and all the complaints generated by excessive heat, by keeping the blood cool and the bowels free with TARRANT'S EFFERVESCENT SELTZER APERIENT, at once a most refreshing draught and the best of all regulating medicines.
SOLD BY ALL DRUGGISTS.

WANTED — AGENTS — $75 to $200 per month, everywhere, male and female, to introduce the GENUINE IMPROVED COMMON SENSE FAMILY SEWING MACHINE. This Machine will stitch, hem, fell, tuck, quilt, cord, bind, braid and embroider in a most superior manner. Price only $18. Fully warranted for five years. We will pay $1000 for any machine that will sew a stronger, more beautiful, or more elastic seam than ours. It makes the "Elastic Lock Stitch." Every second stitch can be cut, and still the cloth cannot be pulled apart without tearing it. We pay Agents from $75 to $200 per month and expenses, or a commission from which twice that amount can be made. Address SECOMB & Co., PITTSBURGH, PA., or BOSTON, MASS.

CAUTION.—Do not be imposed upon by other parties palming off worthless cast-iron machines, under the same name or otherwise. Ours is the only genuine and really practical cheap machine manufactured.

VACCINATION, Genuine German Cow-Pox Virus, fresh from the cow at $2. per Tube. Free by mail. Also WINTON'S VACCINATOR, Silver $3, Gold $5. Sent free by mail on receipt of price. C. S. Halsey, Pharmaceutist, 147 South Clark St. Chicago, Ill., and 238 Main St. Buffalo, N. Y.

### THE OLD SCHOOL-HOUSE.

BY GRANDMOTHER HOWELL.

I remember it, just where it stood at the junction of the two roads shaded by an old knarled apple tree whose ripe, rich fruit was so highly prized by the little school children ; I can see it all now, even to the little brook that ran foaming and fretting on its way, now in the shade, now flashing out in the bright sunshine and so on for about half a mile, when it turned abruptly and fell over a little mound of rocks and emptied into the small pond, or by the more fanciful name of the school children, " Silver Lake." Again I see the old well with the ivy creeping all round it, and the old oaken bucket filled to the brim with clear cold water, so much sweeter than could be found any place else. I see my old friend and ally, Millie, sit ting by my side, with the long black curls half shading the pretty perplexed look, as she bends still lower over the puzzling example. Then when the hour of recess would come, how we would bound out of school, the boys making for the pond, that they might try and sail their miniature ships, and the girls making for the woods in which were hid their precious treasures. Millie and I had a house all to ourselves and we would steal away from the others. The trees formed our roofing and the soft grass our carpet, and with a few pieces of broken crockery and one or two dolls, there were not two happier little girls in all the world. In that one hour we had morning, noon and night; breakfast, and supper. The acorn formed our pretended food, and some of the bright sparkling water mixed with a little dry earth so as to make it brown like chocolate, our pretended drink. I remember how surprised we would be, when the clear silvery sounds of the bell broke in upon our playing. As the afternoon drew to a close, how eagerly we would watch for the long slanting rays of the setting sun to strike through the little window, for then we knew our tasks for that day were done and we had before us the pleasant walk home in the gathering twilight. Sometimes we would make time for a hasty run to our little house to see that all was right, before going home. This was generally on Saturday night, for this was in the old Puritan days, and we would as soon have thought of taking what did not belong to us as to have gone there on Sunday.

And now I change from this bright picture, to the time of our parting. I was to leave the dear old place and go to this great and populous city. The evening before I went, I remember that Millie and I stole down to the dear old schoolhouse for the last time, we sat down on the steps and tried to talk cheerfully of the time when we would visit each other, but in spite of all our efforts, the tears would come; and before we left, we had indulged in a good hearty cry. The quiet walk home I keep sacred in my memory, and now that I am three score years and ten I have a strange fancy to visit the old place, although I know it would be sadly changed in all these years. Oh those were happy golden days in "The Old Red School House."
